# P-629. Exploring the Interaction Between Group A Streptococcal Pneumonia and the COVID-19 Pandemic (GASP)

**DOI:** 10.1093/ofid/ofaf695.842

**Published:** 2026-01-11

**Authors:** Hannah Bray, Felix Bratosin, Adam Caulfield, Zachary Ciochetto, Robert D Fulmer, Marlene Garcia, Jacob Stremers, Gordana Simeunovic

**Affiliations:** Corewell Health/Michigan State University, Muskegon, MI; Corewell Health West/Michigan State University, Grand Rapids, Michigan; Corewell Health, Grand Rapids, Michigan; Corewell Health, Grand Rapids, Michigan; Corewell Health West, Grand Rapids, Michigan; Corewell Health /Michigan State University, Grand Rapids, MI; Corewell Health West/Michigan State University, Grand Rapids, Michigan; Corewell Health/ Michigan State University, grand rapids, Michigan

## Abstract

**Background:**

Group A Streptococcus (GAS) is a known cause of invasive infections (iGAS), with the incidence of GAS pneumonia (GAS PNM) being traditionally low. During the COVID pandemic, the rate of iGAS decreased, followed by a rebound in the years after. Several clusters of GAS PNM were described during this time. Systematic data on GAS PNM epidemiology and outcomes is lacking. In this retrospective study we evaluate the rate of GAS PNM in relation to the pandemic, as well as patient population and outcomes in order to identify risk factors for severe infection.

**Figure:**
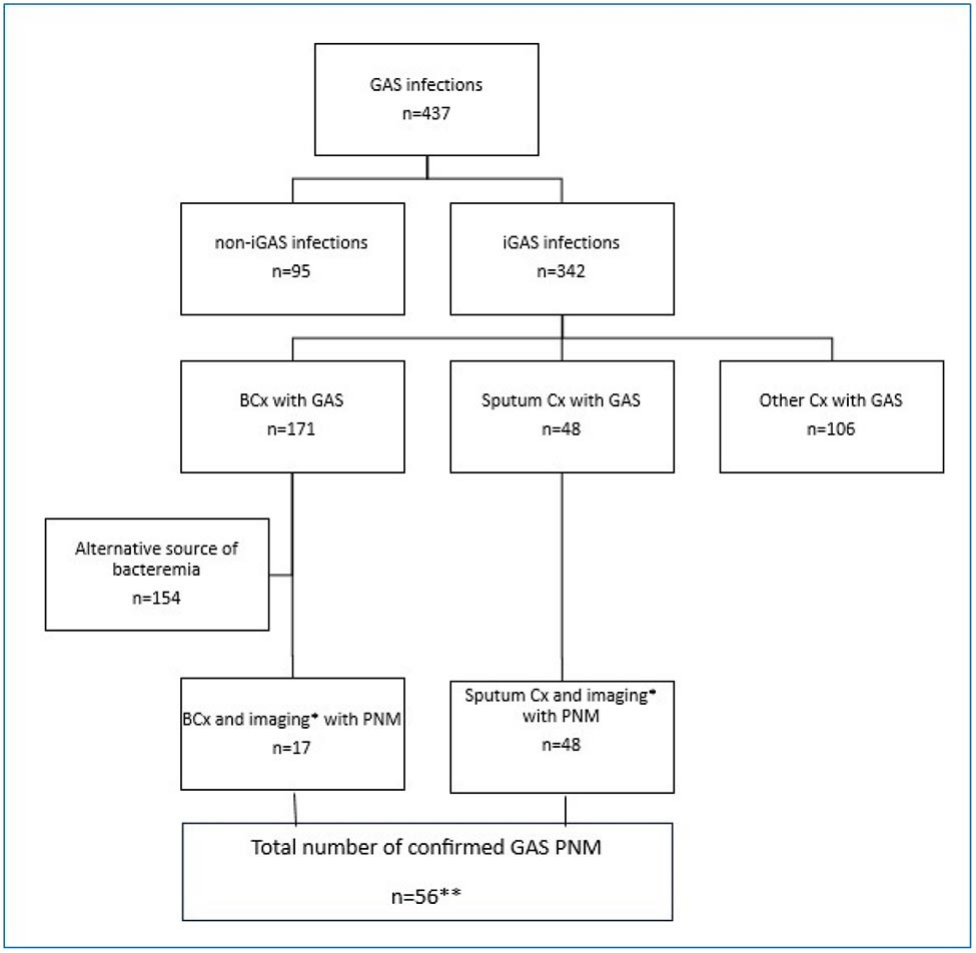
Patients’ recruitment Of all hospitalized patients between April 1, 2018, and March 31, 2024, with positive Group A Streptococcus (GAS) culture, we identified the patients with invasive GAS infection (iGAS) that was defined as a positive culture from any site other than skin and throat. Among patients with iGAS infection, we identified patients with positive sputum cultures and positive blood cultures. Patients with positive blood culture who had an alternative source of bacteremia were excluded by chart review. Remaining patients with positive blood culture and patients with positive sputum culture were reviewed to confirm the presence of pneumonia on imaging, either CT or chest x-ray*, based on radiology reading. If pneumonia was confirmed on imaging, patients were included in the analysis: 11 patients had both positive blood and sputum culture, 37 only sputum culture and 6 only blood culture, making a total of 56 patients**. GAS= Group A Streptococcus infection; iGAS=invasive Group A Streptococcus infection; Bcx=blood cultures; Cx=culture; PNM=pneumonia

**Figure:**
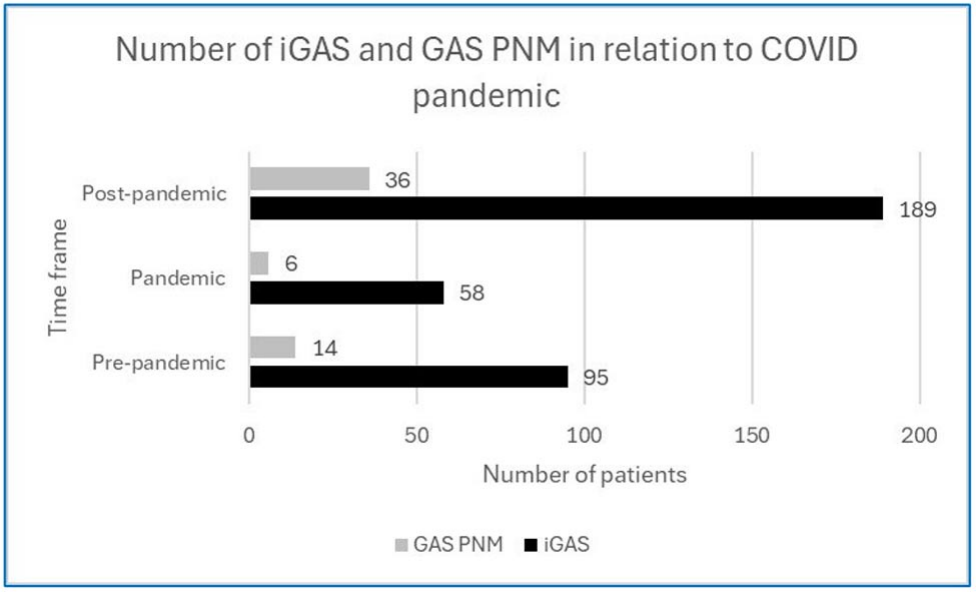
Number of invasive Group A Streptococcus infection (iGAS) and Group A Streptococcus Pneumonia (GAS PNM) in hospitalized patients in relation to COVID pandemic. iGAS infection is defined as a positive culture isolated from any site other than the skin and throat. GAS PNM is defined as pneumonia on imaging (CT and/or chest x-ray) and either positive sputum culture and/or positive blood culture with no other explanation for GAS bacteremia. The evaluated timeframe was divided into pre-pandemic: April 1, 2018, until March 31, 2020); pandemic (April 1, 2020, until March 31, 2022), and post-pandemic (April 1, 2022, until March 31, 2024). The number of both iGAS and GAS PNM increased in post-pandemic compared with pandemic and pre-pandemic period, although the difference was not statistically significant (p=0.48 and p=0.26, respectively).

**Methods:**

Among patients hospitalized between April 1st, 2018, and March 31st, 2024, in Corewell Health West in Michigan, with positive GAS blood and sputum culture, we identified those with GAS PNM (figure 1). A retrospective chart review was conducted to evaluate patients' characteristics and outcomes defined as hospital and ICU length of stay, 90-day all-cause mortality, and readmission rates. The outcomes were evaluated by the time frame (pre-COVID, COVID, and post-COVID) and mortality status using Chi-squared and Fisher exact tests for categorical and Wilcoxon Rank Sum test for numeric variables to assess statistical significance using an alpha of p< 0.05.

**Figure:**
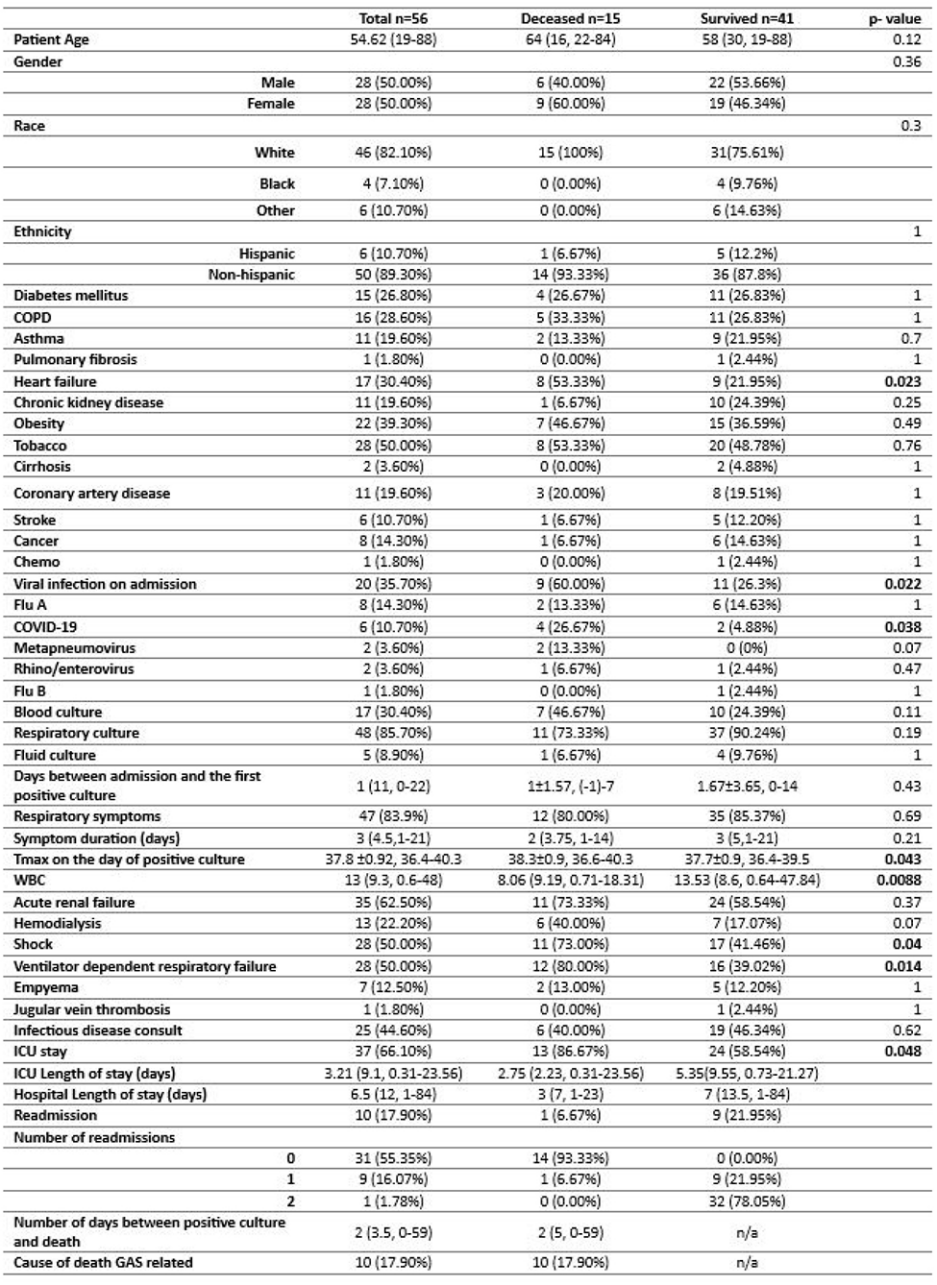
Patient characteristics, presentation, clinical course and outcome of Group A Strep pneumonia (GAS PNA) by 90-day mortality

**Figure:**
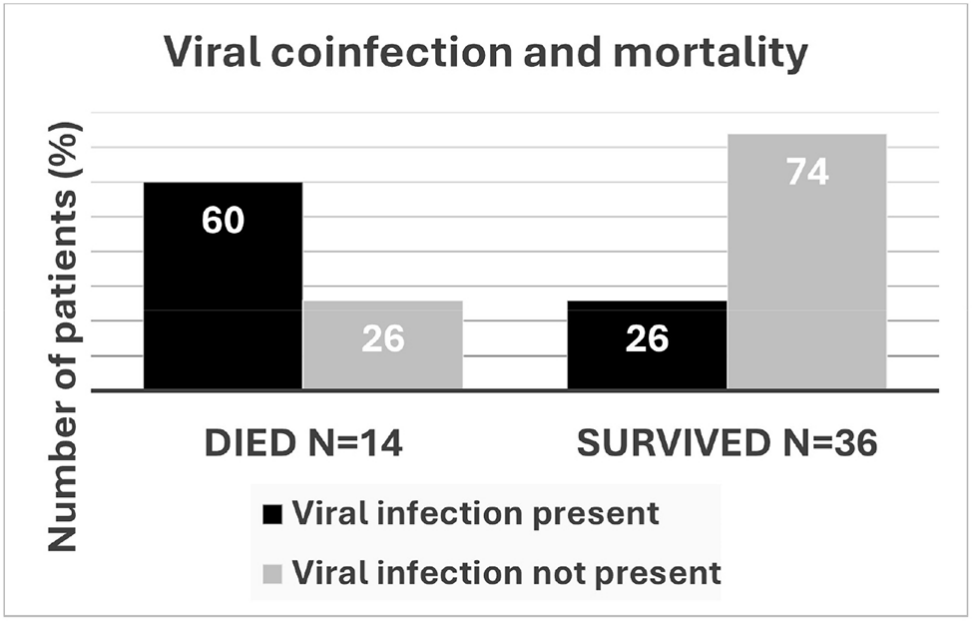
Viral coinfection and mortality. Deceased patients were more likely to have viral coinfection on admission (p=0.022).

**Results:**

The absolute number of GAS PNM increased post-COVID compared with COVID and pre-COVID (Figure 2), but there was no difference in the patients’ characteristics or outcomes among the groups. Overall, 35.7% of patients had a viral coinfection and 66.10% needed ICU level of care with the median ICU stay of 3.21 days (9.1, 0.31-23.56) including 50% of patients requiring ventilator support and 50% of patients experiencing shock requiring vasopressors. 26.8% died within 90 days, with a median time between positive culture and death of 2 days (3.5, 0-59). Deceased patients were more likely to have viral coinfection (p=0.022) and lower WBC (0.0088), and to be febrile (p=0.043) (Figure 3).

**Conclusion:**

Our data suggests GAS PNM is a severe, rapidly progressive disease, especially with viral coinfection with many patients requiring ICU care with complications including acute renal failure, shock, ventilator dependent respiratory failure, and even death. Recognizing disease early and identifying risk factors for severe disease is vital to improving outcomes.

**Disclosures:**

All Authors: No reported disclosures

